# Involvement of inflammatory gene expression pathways in depressed patients with hyperphagia

**DOI:** 10.1038/s41398-019-0528-0

**Published:** 2019-08-20

**Authors:** Hilde de Kluiver, Rick Jansen, Yuri Milaneschi, Brenda W. J. H. Penninx

**Affiliations:** 1 Amsterdam UMC, Vrije Universiteit, Department of Psychiatry, Amsterdam Public Health research institute and Amsterdam Neuroscience, Oldenaller 1, 1081 HJ Amsterdam, the Netherlands; 2 Amsterdam UMC, Vrije Universiteit, Department of Psychiatry, Amsterdam Neuroscience, Oldenaller 1, 1081 HJ Amsterdam, the Netherlands

**Keywords:** Genetics, Genomics

## Abstract

The pathophysiology of major depressive disorder (MDD) is highly heterogeneous. Previous evidence at the DNA level as well as on the serum protein level suggests that the role of inflammation in MDD pathology is stronger in patients with hyperphagia during an active episode. Which inflammatory pathways differ in MDD patients with hyperphagia inflammatory pathways in terms of gene expression is unknown. We analyzed whole-blood gene expression profiles of 881 current MDD cases and 331 controls from the Netherlands Study of Depression and Anxiety (NESDA). The MDD patients were stratified according to patients with hyperphagia (characterized by increased appetite and/or weight, *N* = 246) or hypophagia (characterized by decreased appetite and/or weight, *N* = 342). Using results of differential gene expression analysis between controls and the MDD subgroups, enrichment of curated inflammatory pathways was estimated. The majority of the pathways were significantly (FDR < 0.1) enriched in the expression profiles of MDD cases with hyperphagia, including top pathways related to factors responsible for the onset of inflammatory response (‘caspase’, ‘GATA3’, ‘NFAT’, and ‘inflammasomes’ pathways). Only two pathways (‘adaptive immune system’ and ‘IL-8- and CXCR2-mediated signaling’) were enriched in the MDD with hypophagia subgroup, these were also enriched in the total current MDD group and the group with hyperphagia. This confirms the importance of inflammation in MDD pathology of patients with hyperphagia, and suggests that distinguishing more uniform MDD phenotypes can help in finding their pathophysiological basis.

## Introduction

Major depressive disorder (MDD) is one of the leading causes of morbidity worldwide with a high socioeconomic burden^1,2^. MDD is a heterogeneous disorder for which many etiological factors have been suggested, for instance low-grade inflammatory activation^3,4^. Meta-analyses of peripheral inflammatory marker studies report elevated levels of C-reactive protein (CRP), interleukin(IL)-6, tumor necrosis factor (TNF)-α, the soluble IL-2 receptor and lowered levels of interferon(IFN)-γ in MDD patients^5–8^ compared to controls. For some inflammatory markers, large between-study heterogeneities and small to moderate effect sizes were observed in these meta-analyses, which may partially be attributable to the phenotypic heterogeneity of MDD.

Previous evidence^9–12^ suggests that the link with inflammation is stronger, or even specific, for MDD patients with some of the so-called “reversed neurovegetative symptoms”, such as hyperphagia, weight gain, hypersomnia and fatigue. Recent results from the Netherlands Study of Depression and Anxiety (NESDA) on ~800 MDD patients showed that among reversed neurovegetative symptoms ‘increased appetite’ was the most strongly associated with CRP and TNF-α levels^13^. In line with this are the results of Simmons et al.^14^, who subgrouped 53 current MDD patients solely on direction of appetite change and found elevated levels of IL-1RA, IL-6 and CRP levels in patients with increased appetite and no differences between patients whose appetite is decreased as compared to controls.

Results from genetic studies displayed similar patterns. In a recent genetic study based on >25,000 samples from the Psychiatric Genomics Consortium, MDD cases were stratified according to symptoms of appetite and/or weight change during the index episode: increased appetite and/or weight (15.8%) vs. decreased appetite and/or weight (45.2%). Results showed that the genetic overlap between MDD and CRP levels was enhanced in MDD cases with increased appetite and/or weight, who carried a significantly higher number of risk variants for CRP levels. No associations were found for MDD cases with decreased appetite and/or weight^15^.

At the transcriptome level, several case-control studies have been carried out. In NESDA, 129 differentially expressed genes in current MDD cases were found that were enriched for several pathways among which downregulation of the natural killer cell mediated cytotoxicity pathway and upregulation of IL-6 mediated signaling pathways^16^. Another study reported that 90 upregulated genes were significantly enriched for pathways related to response to infection and innate immune system and 75 downregulated genes were significantly enriched for pathways related to T-cell functioning and the adaptive immune system^17^. Hori et al.^18^ detected 317 differentially expressed genes in which the synaptic transmission pathway was over-represented. Mostafavi et al.^19^ analyzed RNA sequence data and did not find differentially expressed genes between recurrent MDD patients and controls. However, enrichment analyses yielded enrichment of the interferon α/β-signaling pathway. The Young Finns Study carried out immunological and inflammatory processes gene set enrichment analysis in genome-wide expression profiles in participants with a chronic, high-severity depression score as compared to other participants from this population-based cohort^20^. Enriched pathways included IL-1R mediated signaling events, Toll-like receptor pathway and Ras signaling pathway.

As opposed to studies examining the DNA and serum protein level, no gene expression studies have examined potential differential patterns for MDD subtypes so far. The aim of this study was to examine enrichment of inflammatory pathways in gene expression profiles of subgroups of current MDD cases stratified according to symptoms of change in appetite and/or weight. We hypothesize that the more prominent role of inflammation in the MDD with hyperphagia subgroup is also seen at the level of gene expression, and aim at further specifying the inflammatory pathways that could be involved at gene expression level.

## Methods

### Study sample

Participants were part of the NESDA, an ongoing longitudinal cohort study into the long-term course and consequences of depressive and anxiety disorders. A description of the study rationale, design, and methods is given elsewhere^21^. Briefly, from 2004 to 2007, 2981 participants between the ages of 18 and 65 were recruited from the community (19.0%), primary care (54.0%), and specialized mental health care settings (27.0%). These participants were healthy controls or had a current or prior history of depressive and/or an anxiety disorder. Participants were not included when they had a primary diagnosis of bipolar, psychotic, obsessive compulsive or severe addictive disorder and did not speak fluently Dutch. The Ethical Committee of all participating universities approved the NESDA project and all participants provided written informed consent.

Data collection included an extensive interview, blood collection, self-reported questionnaires and medical assessments. For the current study, we selected an analytical sample of 1847 participants with data on MDD diagnostic status and gene expression.

### MDD status and stratification

A Diagnostic and Statistical Manual of Mental Disorders (DSM)-IV MDD diagnosis was established with the use of the composite interview diagnostic instrument (CIDI)—lifetime version 2.1^22^. The analytical sample included 881 patients with current MDD (i.e., diagnosed with major depression in the 6 months prior to baseline), 635 participants with remitted MDD (i.e., lifetime diagnosis of MDD but not current) and 331 healthy controls (i.e., participants without a history of MDD and/or an anxiety disorder).

The current MDD group was stratified according to change in appetite and/or weight during a major depressive episode consistently with previous studies^15,23^. MDD symptom-level data ascertained by the CIDI interview for the most severe episode in lifetime were retrieved. Data on symptoms of change in appetite and were disaggregated to code separately for increase, decrease and both increase/decrease. Cases were assigned “increased” or “decreased” in appetite and/or weight when an increase or decrease in at least one of the symptoms was observed. The other symptom was in the same direction of change, no change of both increase and decrease. This resulted in three subgroups; 246 MDD patients with increased appetite and/or weight (27.9%), 341 with decreased appetite and/or weight (38.7%), and a third group comprising 294 participants (33.4%) who reported no change, both directions or opposite directions in appetite and/or weight change (see Supplementary Table 1 summarizing the steps in stratification). Since previous research indicated that the direction of change in appetite is the most discriminating symptom in terms of inflammatory alterations^13^, and since change in weight commonly parallels those in appetite, we labeled the MDD subgroup with increased appetite and/or weight “MDD with hyperphagia” (85.8% of the cases experienced increase in appetite), and the MDD subgroup with decreased appetite and/or weight “MDD with hypophagia” (88.3% of the cases experienced decrease in appetite). In summary, the stratification of MDD cases is based on appetite change and/or weight change.

Note that our stratification procedure based on symptoms of appetite and/or weight increase and decrease has similarities with the DSM specifier of respectively the atypical and melancholic subtypes regarding these individual symptoms^24^. However, these are not interchangeable since we have not taken the core symptoms of mood reactivity and anhedonia and other reversed neurovegetative symptoms into account. Experiencing appetite and/or weight change are not prerequisites for having the atypical or melancholic depression diagnosis. Since previous evidence suggests that heightened inflammation is not specific for the entire atypical subtype of MDD but rather individual atypical-like symptoms^13^, we have applied stratification only on endorsement of these two symptoms, like has been done previously^15,23^.

The present paper focused a priori on the examination of the current MDD with hyperphagia and the current MDD with hypophagia subgroups, which have been previously shown to have similar higher severity but divergent clinical, biological, and genetic profiles^13,15,23^. Participants reporting no appetite and/or weight change have been shown instead to have low symptoms severity^15^. Participants with remitted MDD were not stratified because remitted MDD have been previously shown to have smaller gene expression differences from controls, as opposed to the current MDD group^16^. Also, there was no difference seen in circulating CRP concentrations, TNF-α and IL-6 levels between remitted MDD patients and controls^25^. For these reasons the main results reported concerns only the two hyper- and hypophagia subgroups and the overall current MDD group considered as benchmark. Nevertheless, the model of the differential gene expression analysis appropriately included all four MDD (sub)groups ((1) remitted MDD patients, (2) current MDD patients with hyperphagia, (3) current MDD patients with hypophagia, and (4) current MDD patients without hyperphagia and hypophagia). Results for the subgroup of patients without hyperphagia and hypophagia, and remitted MDD, are not further discussed.

### Other characteristics

Use of medication was based on drug container inspection of all medications used in the past month, classified according to the World Health Organization Anatomical Therapeutic Chemical (ATC) classification; Use of anti-inflammatory medication (M01A, M01B, A07EB, A07EC) and antidepressant medication (N06AA, N06AB, N06AX) were assessed. Depression severity scores were assessed with the Inventory Depressive Symptomatology (IDS)^26^. This 30-item self-rated questionnaire assesses the presence of all symptom domains of a major depressive episode in the past seven days on a 0–3 scale (not severe–severe). Finally, BMI (in kg/m^2^) and current smoking status at baseline were also measured.

Differences in baseline characteristics between (1) all current MDD cases and controls and (2) the hyper- and hypophagia subgroups and controls were tested using *χ*^2^-tests or analyses of (co)variance (followed by Tukey’s test for pairwise comparisons) were performed.

### Gene expression measurement and analyses

Serial venous samples were obtained (8–10 am, after overnight fasting) in one 7-mL heparin-coated tube (Greiner Bio-One, Monroe, North Carolina). Between 10 and 60 min after blood draw, 2.5 mL of blood was transferred into a PAXgene tube (Qiagen, Valencia, California). This tube was kept at room temperature for a minimum of 2 h and then stored at −20 °C. Total RNA was extracted at the VU University Medical Center (Amsterdam) according to the manufacturer’s protocol (Qiagen). Gene expression in peripheral venous blood was assayed at RUCDR Infinite Biologics (http://www.rucdr.org). Samples were randomized to plates and gene expression profiles were determined using Affymetrix U219 arrays (96-well format) and the GeneTitan System as per the manufacturer’s protocol (see Jansen et al.^16^ for further details). Expression values were obtained using RNA normalization implemented in Affymetrix Power Tools (APT, v 1.12.0). Gene expression levels were measured with 45 574 probe sets and was summarized to gene level by calculating the mean expression level of the probe sets targeting the same gene. This resulted in 18,364 genes available for analysis. Gene expression analyses were corrected for several covariates (age, sex, smoking status, RNA measurement plate, RNA quality measure, RNA measurement well, blood draw lab, days from blood extraction to RNA hybridization, red blood cell count, gene expression-DNA mismatch, hour of blood extraction, month of blood extraction; For each of the 18,364 genes, a linear model was fit with gene expression level as the dependent variable and MDD status as the independent variable, while including the mentioned covariates as independent variables as well. Correction for multiple testing was done according to the Benjamini-Hochberg procedure^27^. These data were used to generate profiles of differentially expressed genes, as compared to healthy controls, of MDD (sub)groups to be used as input for pathway enrichment analyses. While the statistical power to detect single genes differentially expressed at genome-wide significant level may be substantially reduced after stratification, the joint effect of genes in a pathway could be detected more reliably.

### Selection of inflammatory pathways

We identified inflammatory pathways involving cytokines by a search of the curated canonical pathways indexed by the Molecular Signature Database (MSigDB; http://software.broadinstitute.org/gsea/msigdb/search.jsp), limited to Homo sapiens. At the time of search (December 2017), MSigDB contained 1329 canonical pathways. First, an initial search was done using the MSigDB search engine, with the following key words: “inflammation OR inflammatory OR cytokine OR cytokines OR interleukin OR interleukins OR IL OR interleukin-* OR chemokine OR chemokines OR interferon OR IFN* OR interferons OR TNF OR NLRP*”. This resulted in 164 pathways. Second, pathways were removed when there were no cytokines involved and when the pathway concerned a specific disease. A total number of 19 pathways were removed, which are listed in Supplementary Table 2. Finally, 17 inflammatory pathways involving cytokines were not tagged by the key words and were therefore included. The final selection includes 162 pathways, containing 3460 unique genes, from multiple online pathway databases (63 from BioCarta, 23 from KEGG, 31 from Reactome, 36 from PID, and 9 from others), which are listed in Supplementary Table 3. Note that the 162 tested pathways are not distinct pathways since they are originating from different databases and the genes within these pathways overlap to some extent. The final number of significantly enriched pathways is rather a reflection of the strength of involvement of inflammation than the exact pathways involved.

### Pathway enrichment analysis

The *P* values generated in the differential gene expression analyses were used as input for enrichment analyses, for each group separately (all MDD, MDD with hyperphagia and MDD with hypophagia). For each of the 162 inflammatory pathways, we performed a one-sided Wilcoxon rank-sum test, which does not assume a known distribution and tests for a location shift between the *P* values inside the pathway and the *P* values outside the pathway. We purposely included all genes in the pathway enrichment analyses, regardless of the significance level of the genes in the differential gene expression analyses. This method is particularly useful because it includes subthreshold signal; if non-significantly differentially expressed genes belonging to one pathway are relatively clustered towards a higher ranking in *P* values compared to remaining genes, enrichment signal could be noticeable. Correction for multiple testing was performed using the Benjamini-Hochberg procedure^27^. Subsequently, we verified whether the enrichment results were confirmed after estimation of permutation-based FDRs. If correlations between gene expression patterns across different pathways have led to false positives, the number of pathways with an FDR < 0.1 is lower when the permutation-based FDR is applied. This was applied as follows: For each of the 1000 permutations, the *P* values obtained from differential gene expression were permuted among gene, and enrichment analysis was performed using the permuted *P* values. For each observed *P* value from the real observed pathway enrichment analyses a corresponding FDR was estimated. This was done by dividing the average number of *P* values obtained from the permutation-based enrichment analyses lower than the observed *P* value per permutation by the total number of observed *P* values lower than the observed *P* value.

Finally, in order to check whether gene expression in inflammatory pathways has a major role compared to other pathways, we also performed enrichment analyses including all 1329 canonical pathways present in the MSigDB. These 1329 canonical pathways concern inflammatory as well non-inflammatory pathways.

### BMI, inflammation, antidepressant use and MDD with hyperphagia

Since MDD with hyperphagia patients had higher BMI as compared to all other participants, the enrichment of the 162 inflammatory pathways in this subgroup was re-estimated after additional BMI adjustment of the gene expression analysis. Previous studies consistently identified significant genetic correlations between MDD and BMI, indicating potential shared etiological mechanisms^15,28^. Intriguingly, the genetic overlap with BMI has been shown to be especially strong in MDD with hyperphagia^15^. This strong connection at the genetic level is also reflected at the phenotype level^13^. Furthermore, analyses based on Mendelian Randomization principles suggested that BMI is causal for MDD, or BMI is genetically related with a causal factor for MDD^28,29^. So, inflammation may represent an important factor in a causal pathway with obesity as a general underlying route. Thereby, adjusting for BMI may result in overadjustment of the associations between inflammation and depression. As an alternative, examining to what extent BMI plays an explaining role seems more appropriate.

Finally, it is suggested that some antidepressants exert an anti-inflammatory effect^30,31^. In order to check whether antidepressant use is of influence, we have again performed the differential gene expression analysis with exclusion of cases using antidepressant medication.

All statistical analyses were performed in R software version 3.4.1^32^.

## Results

The sample about which is reported below consisted of 881 patients with current MDD and 331 healthy controls (65.3% females, mean age 41.1). Stratification of the current MDD group according to change in appetite and/or weight during a major depressive episode resulted in 246 participants with MDD hyperphagia and 341 participants with MDD with hypophagia. As compared to controls, MDD cases were more likely to be female, younger, current smokers, and to use antidepressant and anti-inflammatory medications, and had a higher IDS score. The subgroup MDD with hyperphagia contained significantly more females, less current smokers, and had a significantly higher BMI than the subgroup MDD with hypophagia. Age and IDS score did not differ between these subgroups (Table 1).

**Table 1:** Sample characteristics (*N* = 1212)

	Healthy controls	all MDD	*P* value (all MDD vs. controls)	MDD with hyperphagia	MDD with hypophagia	*P* value (overall)
N	331	881		246	341	
Female, N (%)	199 (60.1)	593 (67.3)	<0.05^a^	185 (75.2)^d^	228 (66.9)^d^	<0.001^a^
Age, mean (sd.)	42.6 (14.6)	40.6 (12.2)	<0.05^b^	41.1 (11.7)	40.0 (12.1)^e^	<0.05^c^
BMI, mean (sd.)	25.5 (4.7)	25.9 (5.4)	0.18^b^	28.4 (6.0)^d,e^	24.4 (4.9)^d,e^	<0.0001^c^
IDS, mean (sd.)	8.2 (7.4)	32.3 (12.3)	<0.0001^b^	33.8 (11.6)^e^	32.4 (13.3)^e^	<0.0001^c^
Current anxiety diagnosis, *N* (%)	0 (0)	582 (66.1)	<0.0001^a^	170 (69.1)	221 (64.8)	<0.0001^a^
Anti-inflammatory drug use, *N* (%)	6 (1.8)	42 (4.8)	<0.05^a^	14 (5.7)	14 (4.1)	<0.05^a^
Current smoking, *N* (%)	90 (27.2)	406 (46.1)	<0.0001^a^	84 (34.1)^d^	199 (58.4)^d^	<0.0001^a^
Antidepressant medication use, *N* (%)	2 (0.6)	398 (45.2)	<0.0001^a^	103 (42.9)	157 (46.0)	<0.0001^a^

*P* value in the fourth column is for testing difference between total current MDD group and controls. *P* value in the seventh column is for testing differences between the MDD subgroups with increased and decreased appetite and/or weight change and controls and posthoc pairwise comparison

*BMI* body mass index, *IDS* inventory of depressive symptomatology, *MDD* major depressive disorder

^a^*χ*^2^-test

^b^Two-sided *t*-test

^c^ANOVA

^d^Different from the other appetite and/or weight change subgroup

^e^Different from controls

### Inflammatory pathway enrichment analyses

Using genome-wide whole-blood gene expression measurements, we performed differential gene expression analysis between all patients with current MDD, MDD with hyperphagia, and MDD with hypophagia vs. controls. There were 50, 263, and 308 differentially expressed genes (FDR < 0.1) in MDD with hyperphagia subgroup, MDD with hypophagia subgroup and the total current MDD group, respectively. Supplementary Table 4 shows the results of the differential gene expression analysis. Overlap of differentially expressed genes between the groups is shown in the Venn diagram in Supplementary Fig. 1. While the difference in the number of significantly differentially expressed genes may be due to the different sample size of the subgroups, the strength and the direction of effects across subgroups were highly consistent (*r* = 0.77; except for three genes, the directions of the effects were all the same between the two MDD subgroups).

All the *P* values generated in the differential gene expression analysis were used as input for ‘genome-wide’ enrichment analyses. For 162 inflammatory pathways, we tested whether the median *P* value was lower than the median *P* value of the genes outside the pathway (“Methods”). Most pathways (*n* = 32, at FDR < 0.1) were enriched in the MDD with hyperphagia subgroup, including pathways related to inflammasomes, apoptosis and cytokine signaling, namely IL-2, IL-3, IL-4, IL-5, IL-6, IL-8, IL-12, and IL-22. The most strongly enriched pathways are the ‘PID caspase pathway, ‘BioCarta GATA3 pathway’, and ‘IL-3, IL-5 and GM-CSF signaling pathway’. Only two pathways were enriched for the MDD with hypophagia subgroup (i.e., ‘PID IL-8 CXCR2 pathway’ and ‘Reactome adaptive immune system’), and fourteen pathways for the overall current MDD group. The most strongly enriched pathways for the overall current MDD group are ‘KEGG natural killer cell mediated cytotoxicity’, ‘KEGG T cell receptor signaling pathway’ and ‘PID IL-8 CXCR2 pathway’. At a stricter FDR of <0.05, 21 pathways were enriched in the MDD with hyperphagia subgroup, none in the MDD with hypophagia subgroup and ten in the overall current MDD group.

Table 2 presents 35 out of the 162 pathways that are significantly enriched in at least one MDD (sub)group (FDR < 0.1) and *P* values obtained from these analyses. Enrichment analyses were repeated after an additional permutation-based correction, taking into account correlations between gene expression profiles of pathways, providing substantially overlapping results. Only the results of the enrichment analysis of all MDD cases vs. controls differed. The ‘Reactome NLRP3 inflammasome pathway’ reached the significance threshold in in the permutation-based analyses.

**Table 2:** 35 enriched pathways at FDR < 0.1 in at least one group of MDD patients as compared to controls in enrichment analyses of inflammatory pathways

	*P* value		
Pathway	All MDD	MDD with hyperphagia	MDD with hypophagia
KEGG natural killer cell mediated cytotoxicity	**7.94E-6**	**5.20E-4**	0.0138
KEGG T cell receptor signaling	**1.39E-4**	**2.09E-3**	0.0411
PID IL-8 CXCR2	**1.39E-4**	**2.94E-3**	**5.79E-4**
Reactome adaptive immune system	**3.70E-4**	**4.25E-3**	**4.98E-4**
Reactome immune system	**5.60E-4**	**2.07E-3**	3.98E-3
PID IL-8 CXCR1	**5.71E-4**	**6.19E-3**	2.32E-3
Reactome signaling by interleukins	**6.93E-4**	**1.22E-3**	0.0344
PID CXCR4 pathway	**1.41E-3**	**4.80E-3**	7.66E-3
Reactome IL-3, IL-5 and GM-CSF signaling	**2.80E-3**	**4.70E-4**	8.10E-3
Reactome apoptosis	**4.85E-3**	**0.0147**	0.0101
Reactome inflammasomes	**6.08E-3**	**1.71E-3**	0.149
BioCarta natural killer cells pathway	**2.62E-3**	0.0637	4.17E-3
PID aMb2 neutrophils pathway	**7.69E-3**	0.0230	9.63E-3
PID CD8 TCR downstream pathway	**8.42E-3**	0.0283	0.0380
PID caspase pathway	0.0188	**3.76E-4**	0.0558
BioCarta GATA3 pathway	0.0280	**4.10E-4**	0.0359
PID NFAT transcription factor pathway	0.0297	**5.10E-4**	0.154
BioCarta PAR1 pathway	0.203	**1.73E-3**	0.127
Reactome the NLRP3 inflammasome	0.0101	**3.06E-3**	0.0240
BioCarta IL22BP pathway	0.0749	**4.11E-3**	7.54E-3
KEGG apoptosis	0.0452	**5.10E-3**	0.169
PID IL-4 2pathway	0.474	**5.48E-3**	0.170
ST interleukin 4 pathway	0.110	**5.67E-3**	0.0101
Reactome IL-6 signaling	0.143	**6.13E-3**	0.107
PID angiopoietin receptor pathway	0.0136	**6.29E-3**	0.0284
Reactome nucleotide-binding domain leucine-rich repeat containing receptor NLR signaling pathways	0.0520	**7.73E-3**	0.161
Reactome cytokine signaling in immune system	0.0129	**8.36E-3**	0.128
BioCarta IL2RB pathway	0.123	**0.0125**	0.208
BioCarta mitochondria pathway	0.303	**0.0155**	0.224
BioCarta IL-6 pathway	0.497	**0.0162**	0.182
BioCarta TNFR1 pathway	0.389	**0.0167**	0.403
KEGG JAK-STAT signaling pathway	0.173	**0.0173**	0.322
Reactome extrinsic pathway for apoptosis	0.0147	**0.0174**	0.308
PID IL-2 STAT5 pathway	0.137	**0.0181**	0.180
PID IL-12 2pathway	0.0206	**0.0190**	0.0372

The values represent the raw *P* values obtained from the enrichment analyses. Bold values correspond to an FDR < 0.1

The raw *P* values and corresponding FDRs of the enrichment analyses of all 162 inflammatory pathways are listed in Supplementary Table 5. Supplementary Table 6 presents, of the 25% percentile genes with the lowest *P* value within each pathway, the percentage of genes with a positive effect size in the differential gene expression analysis

*CD* cluster of differentiation, *CXCR* chemokine receptor, *GM-CSF* granulocyte-macrophage colony-stimulating factor, *IL* interleukin, *IL22BP* interleukin-22 binding protein, *IL2RB* interleukin-2 receptor beta subunit, *JAK* Janus Kinase, *KEGG* Kyoto Encyclopedia of Genes and Genomes, *NFAT* nuclear factor of activated T cells, *NLR* nucleotide-binding domain leucine-rich repeat, *NLRP3* nucleotide-binding domain leucine-rich repeat containing family pyrin domain-containing-3, *PAR1* protease-activated receptors, *ST* signaling transduction KE, *STAT* signal transducer and activator of transcription, *TCR* T cell receptor, *TNFR1* tumor necrosis factor receptor 1

**Fig. 1:**
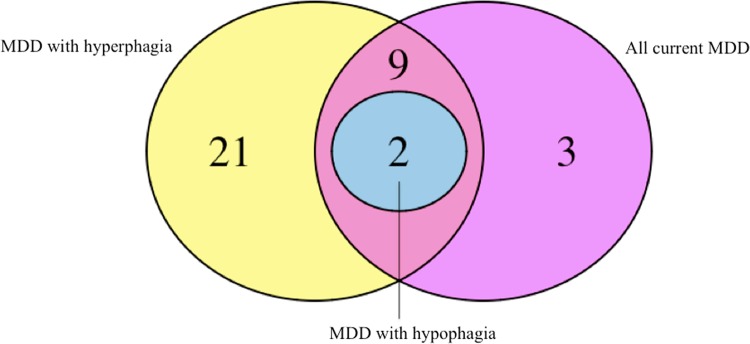
Number of enriched inflammatory gene expression pathways per group at FDR < 0.1

The Venn diagram in Fig. 1 shows the overlap between the enriched pathways at an FDR < 0.1 in the three groups of MDD patients. In general, it could be said that inflammatory pathways were more strongly involved in the MDD subtype endorsing hyperphagia. There are 21 inflammatory pathways uniquely enriched at FDR < 0.1 in the MDD with hyperphagia subgroup, which are listed in the lower 21 rows of Table 2. Noteworthy, among these pathways are two pathways on IL-6 mediated signaling, two on IL-4 mediated signaling and four apoptosis-related pathways (i.e., ‘PID caspase pathway’, ‘KEGG apoptosis’, ‘BioCarta mitochondria pathway’, ‘Reactome extrinsic pathway for apoptosis’). The majority of pathways enriched in the total MDD group were also enriched in the MDD with hyperphagia subgroup. Only three pathways (i.e., downstream signaling of naïve CD8+ T cells, aMb2 integrin, and natural killer cells) were uniquely enriched when considering together all current MDD cases. The only two enriched pathways in MDD cases with hypophagia (i.e., adaptive immune system and IL-8 CXCR2 signaling pathway) were also enriched in both analyses of the subgroup of MDD with hyperphagia and the total current MDD group.

### Enrichment analyses using all canonical pathways

Enrichment analyses using all 1329 canonical pathways were performed to test whether inflammation pathways indeed show up importantly as compared to the other canonical pathways. Supplementary Table 7 shows the 32 out of the 1329 pathways that are significantly enriched in at least one MDD (sub)group as compared to controls (FDR < 0.1). For the total MDD group, 27 pathways were enriched, of which 9 were part of the 162 inflammatory pathways we initially selected. Similar to the main enrichment analysis, the most strongly associated pathway was the ‘KEGG natural killer cell mediated cytotoxicity’. Note that the expression profiles of the total group of current MDD cases are enriched for many pathways regarding T cell signaling. The majority of canonical pathways enriched for the MDD with hyperphagia subgroup (five out of nine) were inflammatory pathways. The most strongly enriched pathway for this subgroup is ‘BioCarta caspase pathway’, which was not selected as an inflammatory pathway in the main analysis. The top pathways in the main enrichment analysis did survive the multiple testing penalty in this analysis including all canonical pathways. No enriched pathway was seen in MDD with hypophagia. These results confirm that even when tested together with all kinds of different biological pathways, inflammation still plays a prominent role in the gene expression profiles of the MDD with hyperphagia subgroup.

### BMI, inflammation, antidepressant use, and MDD with hyperphagia

In order to examine the impact of BMI on the association between inflammatory gene expression and MDD with hyperphagia, we repeated for this subgroup the pathway enrichment analysis after correcting the gene expression analysis for BMI.

A relative attenuation of the signals was shown at the gene level as well as at the pathway level. The mean effect sizes of genes that were significantly differently expressed in MDD with hyperphagia as compared to controls were reduced by 13.1% after BMI adjustment. Of the 32 initially enriched pathways in MDD with hyperphagia, 22 were still nominally significant after BMI adjustment. Five pathways were enriched at an FDR < 0.1, namely ‘PID NFAT transcription factor pathway’, ‘PID IL-8 CXCR2 pathway’, ‘PID caspase pathway’, ‘PID CXCR4 pathway’ and ‘BioCarta GATA3 pathway’. Except for ‘Reactome IL-3, IL-5 and GM-CSF signaling’ and ‘KEGG natural killer cell mediated cytotoxicity’, the most strongly associated pathways in the inflammatory pathways enrichment analysis were also enriched after BMI adjustment. The results of the inflammatory pathway enrichment analysis after BMI adjustment are listed in Supplementary Table 8.

Additional differential gene expression analysis after exclusion of antidepressant users suggests no major influence of its use within the subgroup of MDD with hyperphagia. The beta-beta plot in Supplementary Figs. 2 and 3 show the consistencies between the two differential gene expression analyses. Spearman ρ between the effect sizes of all genes with and without these antidepressant using cases was 0.75 and 1 of the significantly differentially expressed genes (*n* = 6 at FDR < 0.1). This is in line with NESDA’s previous transcriptomic study that found no effect of antidepressant use in expression profiles of currently depressed cases^16^.

## Discussion

This study evaluated enrichment of inflammatory pathways among mean gene expression profiles of two MDD subgroups that can be distinguished based on the direction of appetite and/or weight change (hyperphagia and hypophagia) during an episode, compared to controls. The large majority of enriched inflammatory pathways were found only when focusing on the MDD with hyperphagia subgroup. Almost all the inflammatory pathways that were found to be enriched in the pooled group of patients with MDD were also detected in the MDD with hyperphagia subgroup, despite the substantial reduction in sample size. This suggests that the inflammatory signal in the pooled group of patients with MDD may be driven by this subgroup. These findings complement previous serum proteomic and genetic studies showing, respectively, higher circulating levels of inflammatory markers and increased genetic risk for high CRP levels in MDD patients with hyperphagia^9–12,14,15^. The current study indicates that, also at the transcriptome level, inflammation is especially pronounced in individuals who experience hyperphagia during their current depressive episode. Moreover, we show which inflammatory pathways are most prominent. Conclusions on the directions of this involvement, however, cannot be drawn.

In the MDD with hyperphagia subgroup, there were three pathways associated in the main inflammatory pathway analysis, the inflammatory pathway enrichment analysis after BMI adjustment as well as in the canonical pathway enrichment analysis. These robustly enriched pathways are the ‘BioCarta GATA3 pathway’, ‘PID NFAT transcription factor pathway’, and the ‘PID caspase pathway’. These pathways include several transcription factors (GATA3, NFAT) and enzymes (caspases) that have a major role in the onset of the inflammatory response. For instance, caspase-1 activates the pro-inflammatory cytokines IL-1β and IL-18, by cleaving their precursors. Caspase-1 have been shown to be associated with depression-like behavior in mice before^33,34^ and increased gene expression of its coding gene were observed in blood cells of untreated depressed patients^35^. Caspase-1 is also a downstream target of the inflammasome complex^36^, groups of protein complexes that are triggered by molecular patterns induced by physiological and psychosocial stress. According to the inflammasome hypothesis^36^, psychological stress-induced activation of the NLRP3 inflammasome regulates caspase-1 activation and pro-inflammatory cytokine secretion, which causes subsequent development of depression^36,37^. Interestingly, in our study two pathways related to inflammasomes (‘Reactome inflammasomes’ and ‘Reactome NLRP3 inflammasome’) were strongly enriched in the MDD with hyperphagia subgroup. It is suggested that the NLRP3 inflammasome is involved in comorbid diseases such as obesity^36,38,39^. In mice, targeting the NLRP3 inflammasome has already been successful in reducing depressive-like behavior^40^. To our knowledge, there are no studies suggesting the NLRP3 inflammasome is specifically involved in depression with hyperphagia. Furthermore, caspase-1 is suggested to contribute to glucocorticoid resistance and, subsequently, increases levels of glucocorticoids (e.g., cortisol). These are reflective signs of HPA-axis hyperactivation, one of the most studied biological risk factors associated with MDD^4^. If HPA-axis activation associated with MDD involves caspase-1 in the molecular mechanisms, enrichment of these pathways might relate to that.

Among the pathways uniquely enriched in the MDD with hyperphagia were several linked to signaling of IL-2 or its receptor, IL-4, TNF and IL-6, which is in line with earlier findings showing that IL-6 blood concentrations were especially higher in MDD patients with hyperphagia^13,14^. Other enriched pathways in the MDD with hyperphagia subgroup were related to apoptosis. Increased apoptosis and disrupted proliferation of peripheral mononuclear blood cells in depressed patients compared to controls is previously suggested^41–45^.

There were two enriched pathways in expression profiles of MDD with hypophagia, which were also enriched in the two other groups (i.e., ‘Reactome adaptive immune system’ and ‘PID IL-8 CXCR2 pathway’). Three pathways were uniquely enriched in overall current MDD group (i.e., ‘BioCarta natural killer cells pathway’, ‘PID a_M_b_2_ neutrophils pathway’, and ‘PID downstream signaling of naïve CD8 +T cells’). The latter three may be enriched because of greater power due to the increase in sample size in the total MDD group compared to the MDD subgroups. These five pathways are likely to be generally involved in MDD and are related to the innate immune system as well as the adaptive immune system. The involvement of activation of the innate immune system in MDDs pathogenesis (e.g., pro-inflammatory cytokine secretion) has been widely reported^46^. Innate immune suppression, also in the form of decreased natural killer cell mediated cytotoxicity, has been associated with MDD before^16,45,47^. Impairments in the adaptive immune system in relation to MDD have also been reported^17,44^.

The present results confirm also that BMI may play a role in the relationship between inflammation and MDD with hyperphagia. After adjustment for BMI, the pathway signals were relatively attenuated, although the top pathways (‘PID NFAT transcription factor pathway’, ‘PID IL-8 CXCR2 pathway’, ‘PID caspase pathway’, ‘PID CXCR4 pathway’, and ‘BioCarta GATA3 pathway’) remained significantly associated. The pathways ‘Reactome immune system’, ‘KEGG JAK-STAT signaling pathway’, and ‘KEGG apoptosis’ are found to be significantly enriched in obesity gene expression analyses^48–50^ and were strongly attenuated after BMI correction (FDR: 0.034 to 0.453; 0.094 to 0.426; and 0.049 to 0.203, respectively), indicating that these pathways especially show overlap between obesity and depression.

Rather than a simple confounder, the role of BMI may be described in a more complex interrelation. It has already been reported that especially MDD with increased appetite and/or weight during a depressive episode share genetic risk with BMI as well as inflammatory marker levels^15,28^. Bidirectional Mendelian Randomization analyses between BMI and MDD^28^ and BMI and depressive symptoms^29^ provided evidence for a causality of BMI for MDD and depressive symptoms but not for a reverse causality. In other words, these analyses suggest that BMI is a causal risk factor or associated with causal risk factors for MDD and depressive symptoms. In short, inflammation is a characteristic of both obesity and a subgroup of MDD patients with a higher BMI. It could be hypothesized that a higher BMI may induce a chronic inflammatory state, which, in turn contributes to development of depression symptomatology. The link with this specific symptom may be explained by the negative effect of inflammation on important neuroendocrine regulators of energy homeostasis, such as on the leptin–melanocortin signaling, which regulates food intake and promotes energy expenditure. It is suggested that central inflammation may impair the functioning of leptin receptors expressed in the brain, resulting in leptin resistance that blunts its anorexigenic effect and consequently disinhibits feeding despite increasing circulating leptin (a process comparable with insulin resistance in type 2 diabetes)^38,51^. In line with this hypothesis, high leptin levels have been found specifically in MDD patients with hyperphagia, independently from BMI^52^. Furthermore, MDD patients with hyperphagia have been shown to carry a higher number of genetic risk variants for increased BMI, CRP and leptin levels compared to controls^15^.

In addition to BMI, it could be that MDD with hyperphagia share underlying mechanisms with bipolar disorder. Serum levels of pro-inflammatory cytokines previously associated with MDD were also associated with bipolar disorder^53^. A probabilistic approach^54^ suggested that, among the individuals experiencing a depressive episode without a prior history of manic episodes, individuals with hyperphagia were more probable to have a later diagnosis of bipolar disorder.

The potential causal role inflammation in MDD pathogenesis has stimulated the examination of antidepressant effect of cytokine-inhibiting medication^55,56^. Clinical trials carried out so far reported promising results; overall reductions in depressive symptoms severity after anti-inflammatory treatment were seen in a large meta-analysis^57^, although effects were highly heterogeneous suggesting the presence of subgroups with differential effect size. Also, anti-inflammatory treatment was suggested to be especially beneficial for subgroups of patients marked by elevated inflammation^55–58^. These data underline the need to identify subgroups of patients who may benefit most from an anti-inflammatory treatment according to a personalized medicine approach. The current findings suggest that the subgroup may be marked by symptoms of hyperphagia.

Several limitations of our study should be noted. Analyzing subgroups of current MDD patients have come on cost of power. This may have led to false positives or truly associated pathways that were not detected. However, we have applied permutation-based correction for multiple testing additionally, in order to check for false positives, which yielded similar results. False positives due to correlations between gene expression patterns across different pathways are therefore less likely. Also, whole-blood gene expression was measured. Inflammatory gene expression profiles in other tissues (e.g., relevant brain areas) that are informative about the pathophysiology of MDD might have been different. Finally, we did not correct for cortisol levels or HPA-axis activity. In NESDA, cases marked by hyperphagia had different cortisol levels compared to cases marked by hypophagia. We did not control for cortisol levels since we were mainly interested in the MDD with hyperphagia subgroup, which was found to have comparable cortisol levels to controls^11,59^.

To conclude, inflammatory pathways are enriched in expression profiles of MDD patients with hyperphagia. This study further supports the idea that there is a distinct depressive subtype, with partly differential pathophysiology regarding immuno-metabolic processes. Future research will have to show whether this subgroup benefits most from anti-inflammatory treatment strategies, according to a personalized medicine approach, and system biology approaches will have to shed light on the specific transcriptomic pathways involved and identify genes driving the association.

## Supplementary information

Supplementary Tables 1-3, 5-8

Supplementary Table 4

Supplementary Figure 1: Number of differentially expressed genes per (sub)group of MDD cases at FDR<0.1

Supplementary Figure 2: Beta-beta plot of results of the differential gene expression analyses including and excluding cases using antidepressant medication

Supplementary Figure 3: Beta-beta plot of the 6 differentially expressed genes including and excluding cases using antidepressant medication
