# Supplementary material for: Involvement of inflammatory gene expression pathways in depressed patients with hyperphagia
Source: Transl Psychiatry. 2019 Aug 20;9:193. doi: 10.1038/s41398-019-0528-0 (PMC6702221; doi:10.1038/s41398-019-0528-0)
Supplement: Supplementary file 1 — Supplementary Tables 1-3, 5-8 [file 41398_2019_528_MOESM1_ESM.docx]

**Supplementary Table 1: Cross-tabulation stratification phenotype according to symptoms of appetite and/or weight change during major depressive episode**

**Supplementary Table 2: Pathways obtained from the initial search in MSigDB that are excluded for enrichment**

**Supplementary Table 3: Inflammatory pathways (162) tested in enrichment analyses**

**Supplementary Table 5: Results enrichment analyses of 162 inflammatory pathways per group of MDD patients as compared to controls**

**Supplementary Table 6: Percentage of the 25% percentile genes with a positive effect size in the differential gene expression analysis, for each of the 35 enriched (FDR<0.1) pathway separately**

**Supplementary Table 7: 32 canonical pathways enriched at FDR<0.1 in at least one group of MDD patients as compared to controls in overall enrichment analyses including all 1 329 canonical pathways**

**Supplementary Table 8: Results of enrichment analysis of 162 inflammatory pathways after adjustment for BMI in current MDD patients with hyperphagia as compared to controls**

**Supplementary Table 1: Cross-tabulation stratification phenotype according to symptoms of appetite and/or weight change during major depressive episode**

| *N* | **Weight** |  |  |  |
| --- | --- | --- | --- | --- |
| **Appetite** | no change | decreased | increased | both increased and decreased |
| no change | 239 | 21 | 21 | 2 |
| decreased | 145 | 153 | 9 | 3 |
| increased | 98 | 8 | 105 | 8 |
| both increased and decreased | 19 | 19 | 14 | 17 |

χ^2^=499.6; d.f.=9; *p*<.0001

Participants were classified as follows:

Subgroup with decreased appetite and/or weight, “MDD with hypophagia”: 21+153+19+145+3=341

Subgroup with increased appetite and/or weight, “MDD with hyperphagia”: 98+105+8+21+14=246

Not classified as “decreased” nor as “increased”: 239+19+8+9+2+17=294

**Supplementary Table 2: Pathways obtained from the initial search in MSigDB that are excluded for enrichment**

| **Excluded pathway** | **Description/Reason for exclusion** |
| --- | --- |
| BioCarta HIF pathway | - Gene set activated in hypoxia conditions in heart tissue - No cytokines in gene set |
| BioCarta HIVNEF pathway | - Gene set involved in HIV infection |
| BioCarta Keratinocyte pathway | - Gene set involved in keratinocyte differentiation |
| BioCarta Neurotransmitters pathway | - Gene set involved in biosynthesis of neurotransmitters - No cytokines in gene set |
| BioCarta RANKL pathway | - Involved in bone remodeling |
| BioCarta TC Apoptosis pathway | - Pathway involved in HIV induced T-cell apoptosis |
| KEGG Allograft rejection | - Pathway activated after organ transplant |
| KEGG Alzheimer’s disease | - Alzheimer’s disease pathway |
| KEGG Amyotrophic lateral sclerosis ALS | - ALS pathway |
| KEGG Asthma | - Asthma pathway |
| KEGG autoimmune thyroid disease | - Autoimmune thyroid disease pathway |
| KEGG Dilated cardiomyopathy | - Cardiomyopathy pathway |
| KEGG Graft-versus-host disease | - Specific disease after bone marrow transplantation |
| KEGG Hypertrophic cardiomyopathy HCM | - Cardiomyopathy pathway |
| KEGG Leishmania infection | - Gene set involved after leishmania infection |
| KEGG Systemic lupus erythematosus | - Involved in lupus |
| KEGG Viral myocarditis | - Gene set activated after infection of cardiac muscle by a virus. - No cytokines in pathway |
| PID HIV NEF pathway | - HIV pathway |
| Reactome Developmental biology | - No inflammatory pathway |

**Supplementary Table 3: Inflammatory pathways (162) tested in enrichment analyses**

| **Pathway** | **Description** |
| --- | --- |
| BioCarta 41BB pathway | The 4-1BB-dependent immune response |
| BioCarta asb cell pathway | Antigen Dependent B Cell Activation |
| BioCarta ß-Arrestin Src pathway | Roles of fl-arrestin-dependent Recruitment of Src Kinases in GPCR Signaling |
| BioCarta CCR3 pathway | CCR3 signaling in Eosinophils |
| BioCarta CCR5 pathway | Pertussis toxin-insensitive CCR5 Signaling in Macrophage |
| BioCarta CD40 pathway | CD40L Signaling Pathway |
| BioCarta CDMAC pathway | Cadmium induces DNA synthesis and proliferation in macrophages |
| BioCarta Classic pathway | Classical Complement Pathway |
| BioCarta CXCR4 pathway | CXCR4 Signaling Pathway |
| BioCarta Cytokine pathway | Cytokine Network |
| BioCarta DC pathway | Dendritic cells in regulating TH1 and TH2 Development |
| BioCarta EPO NF-kB pathway | Erythropoietin mediated neuroprotection through NF-kB |
| BioCarta Eryth pathway | Erythrocyte Differentiation Pathway |
| BioCarta FAS pathway | FAS signaling pathway (CD95) |
| BioCarta FCER1 pathway | Fc Epsilon Receptor I Signaling in Mast Cells |
| BioCarta fMLP pathway | fMLP induced chemokine gene expression in HMC-1 cells |
| BioCarta Free pathway | Free Radical Induced Apoptosis |
| BioCarta GATA3 pathway | GATA3 participate in activating the Th2 cytokine genes expression |
| BioCarta GCR pathway | Corticosteroids and cardioprotection |
| BioCarta GH pathway | Growth Hormone Signaling Pathway |
| BioCarta Granulocytes pathway | Adhesion and Diapedesis of Granulocytes |
| BioCarta HSP27 pathway | Stress Induction of HSP Regulation |
| BioCarta IL-10 pathway | IL-10 Anti-inflammatory Signaling Pathway |
| BioCarta IL-12 pathway | IL-12 and Stat4 Dependent Signaling Pathway in Th1 Development |
| BioCarta IL-17 pathway | IL-17 Signaling Pathway |
| BioCarta IL1R pathway | Signal transduction through IL1R |
| BioCarta IL-2 pathway | IL-2 signaling pathway |
| BioCarta IL22BP pathway | IL-22 Soluble Receptor Signaling Pathway |
| BioCarta IL2RB pathway | IL-2 Receptor Beta Chain in T cell Activation |
| BioCarta IL-3 pathway | IL-3 signaling pathway |
| BioCarta IL-4 pathway | IL-4 signaling pathway |
| BioCarta IL-5 pathway | IL-5 Signaling Pathway |
| BioCarta IL-6 pathway | IL-6 signaling pathway |
| BioCarta IL-7 pathway | IL-7 Signal Transduction |
| BioCarta Inflam pathway | Cytokines and Inflammatory Response |
| BioCarta LAIR pathway | Cells and Molecules involved in local acute inflammatory response |
| BioCarta Longevity pathway | The IGF-1 Receptor and Longevity |
| BioCarta Lym pathway | Adhesion and Diapedesis of Lymphocytes |
| BioCarta MAPK pathway | MAPKinase Signaling Pathway |
| BioCarta Mitochondria pathway | Role of Mitochondria in Apoptotic Signaling |
| BioCarta Monocyte pathway | Monocyte and its Surface Molecules |
| BioCarta NF-kB pathway | NF-kB Signaling Pathway |
| BioCarta Nkcells pathway | Ras-Independent pathway in NK cell-mediated cytotoxicity |
| BioCarta NKT pathway | Selective expression of chemokine receptors during T-cell polarization |
| BioCarta NO2 IL-12 pathway | NO2-dependent IL-12 Pathway in NK cells |
| BioCarta NTHI pathway | NFkB activation by Nontypeable Hemophilus influenzae |
| BioCarta p27 pathway | Regulation of p27 Phosphorylation during Cell Cycle Progression |
| BioCarta p38 MAPK pathway | p38 MAPK Signaling Pathway |
| BioCarta PAR1 pathway | Thrombin signaling and protease-activated receptors |
| BioCarta PML pathway | Regulation of transcriptional activity by PML |
| BioCarta PPARa pathway | Mechanism of Gene Regulation by Peroxisome Proliferators via PPARa(alpha) |
| BioCarta RelA pathway | Acetylation and Deacetylation of RelA in The Nucleus |
| BioCarta RNA pathway | Double Stranded RNA Induced Gene Expression |
| BioCarta SODD pathway | SODD/TNFR1 Signaling Pathway |
| BioCarta Stem pathway | Regulation of hematopoiesis by cytokines |
| BioCarta Stress pathway | TNF/Stress Related Signaling |
| BioCarta TALL1 pathway | TACI and BCMA stimulation of B cell immune responses. |
| BioCarta Th1/Th2 pathway | Th1/Th2 Differentiation |
| BioCarta T helper pathway | T Helper Cell Surface Molecules |
| BioCarta Tid pathway | Chaperones modulate interferon Signaling Pathway |
| BioCarta TNFR1 pathway | TNFR1 Signaling Pathway |
| BioCarta Tob1 pathway | Role of Tob in T-cell activation |
| BioCarta Toll pathway | Toll-Like Receptor Pathway |
| KEGG Adipocytokine signaling pathway | Adipocytokine signaling pathway |
| KEGG Antigen processing and presentation | Antigen processing and presentation |
| KEGG Apoptosis | Apoptosis |
| KEGG Chemokine signaling pathway | Chemokine signaling pathway |
| KEGG Complement and coagulation cascades | Complement and coagulation cascades |
| KEGG Cytokine-cytokine receptor interaction | Cytokine-cytokine receptor interaction |
| KEGG Cytosolic DNA-sensing pathway | Cytosolic DNA-sensing pathway |
| KEGG Fc epsilon RI signaling pathway | Fc epsilon RI signaling pathway |
| KEGG Hematopoietic cell lineage | Hematopoietic cell lineage |
| KEGG Intestinal immune network for IgA production | Intestinal immune network for IgA production |
| KEGG JAK-STAT signaling pathway | JAK-STAT signaling pathway |
| KEGG Leukocyte transendothelial migration | Leukocyte transendothelial migration |
| KEGG MAPK signaling pathway | MAPK signaling pathway |
| KEGG Natural killer cell-mediated Cytotoxicity | Natural killer cell mediated cytotoxicity |
| KEGG NOD-like receptor signaling pathway | NOD-like receptor signaling pathway |
| KEGG Proteasome | Proteasome |
| KEGG Regulation of Autophagy | Regulation of autophagy |
| KEGG RIG-I-like receptor signaling pathway | RIG-I-like receptor signaling pathway |
| KEGG T cell receptor signaling pathway | T cell receptor signaling pathway |
| KEGG TGF-beta signaling pathway | TGF-beta signaling pathway |
| KEGG Toll-like receptor signaling pathway | Toll-like receptor signaling pathway |
| KEGG Type I diabetes mellitus | Type I diabetes mellitus |
| KEGG Type II diabetes mellitus | Type II diabetes mellitus |
| NABA matrisome | Ensemble of genes encoding extracellular matrix and extracellular matrix-associated proteins |
| NABA matrisome associated | Ensemble of genes encoding ECM-associated proteins including ECM-affiliated proteins. ECM regulators and secreted factors |
| NABA Secreted Factors | Genes encoding secreted soluble factors |
| PID aMb2 neutrophils pathway | aMb2 Integrin signaling |
| PID Angiopoietin receptor pathway | Angiopoietin receptor Tie2-mediated signaling |
| PID Anthrax pathway | Cellular roles of Anthrax toxin |
| PID AP-1 pathway | AP-1 transcription factor network |
| PID ATF-2 pathway | ATF-2 transcription factor network |
| PID Caspase pathway | Caspase cascade in apoptosis |
| PID CD8 TCR downstream pathway | Downstream signaling in naive CD8+ T cells |
| PID Ceramide pathway | Ceramide signaling pathway |
| PID CXCR3 pathway | CXCR3-mediated signaling events |
| PID CXCR4 pathway | CXCR4-mediated signaling events |
| PID HDAC class I pathway | Signaling events mediated by HDAC Class I |
| PID IFNg pathway | IFN-gamma pathway |
| PID IL-1 pathway | IL-1-mediated signaling events |
| PID IL-12 2pathway | IL-12-mediated signaling events |
| PID IL-12 STAT4 pathway | IL-12 signaling mediated by STAT4 |
| PID IL-2 1pathway | IL-2-mediated signaling events |
| PID IL-2 PI3K pathway | IL-2 signaling events mediated by PI3K |
| PID IL-2 STAT5 pathway | IL-2 signaling events mediated by STAT5 |
| PID IL-23 pathway | IL-23-mediated signaling events |
| PID IL-27 pathway | IL-27-mediated signaling events |
| PID IL-3 pathway | IL-3-mediated signaling events |
| PID IL-4 2pathway | IL-4-mediated signaling events |
| PID IL-5 pathway | IL-5-mediated signaling events |
| PID IL-6 7 pathway | IL-6-mediated signaling events |
| PID IL-8 CXCR1 pathway | IL-8- and CXCR1-mediated signaling events |
| PID IL-8 CXCR2 pathway | IL-8- and CXCR2-mediated signaling events |
| PID NFAT transcription factor pathway | Calcineurin-regulated NFAT-dependent transcription in lymphocytes |
| PID NF-kappaB canonical pathway | Canonical NF-kappaB pathway |
| PID PDGFR-α pathway | PDGFR-alpha signaling pathway |
| PID REG GR pathway | Glucocorticoid receptor regulatory network |
| PID RXR/VDR pathway | RXR and RAR heterodimerization with other nuclear receptor |
| PID SHP2 pathway | SHP2 signaling |
| PID SMAD2/3 nuclear pathway | Regulation of nuclear SMAD2/3 signaling |
| PID TCR calcium pathway | Calcium signaling in the CD4+ TCR pathway |
| PID Telomerase pathway | Regulation of Telomerase |
| PID TNF pathway | TNF receptor signaling pathway |
| Reactome Adaptive immune system | Genes involved in Adaptive Immune System |
| Reactome Antiviral mechanism by IFN-stimulated genes | Genes involved in Antiviral mechanism by IFN-stimulated genes |
| Reactome Apoptosis | Genes involved in Apoptosis |
| Reactome Chemokine receptors bind chemokines | Genes involved in Chemokine receptors bind chemokines |
| Reactome Cytokine signaling in immune system | Genes involved in Cytokine Signaling in Immune system |
| Reactome Extrinsic pathway for apoptosis | Genes involved in Extrinsic Pathway for Apoptosis |
| Reactome Factors involved in megakaryocyte development and platelet production | Genes involved in Factors involved in megakaryocyte development and platelet production |
| Reactome G alpha I signaling events | Genes involved in G alpha (i) signaling events |
| Reactome Hemostasis | Genes involved in Hemostasis |
| Reactome IL-2 signaling | Genes involved in Interleukin-2 signaling |
| Reactome IL-3, IL-5 and GM-CSF signaling | Genes involved in Interleukin-3,- 5 and GM-CSF signaling |
| Reactome IL-6 signaling | Genes involved in Interleukin-6 signaling |
| Reactome IL-7 signaling | Genes involved in Interleukin-7 signaling |
| Reactome IL receptor SHC signaling | Genes involved in Interleukin receptor SHC signaling |
| Reactome IL-1 signaling | Genes involved in Interleukin-1 signaling |
| Reactome Immune system | Genes involved in Immune System |
| Reactome Inflammasomes | Genes involved in Inflammasomes |
| Reactome Innate immune system | Genes involved in Innate Immune System |
| Reactome Interferon alpha/beta signaling | Genes involved in Interferon alpha/beta signaling |
| Reactome Interferon gamma signaling | Genes involved in Interferon gamma signaling |
| Reactome Interferon signaling | Genes involved in Interferon Signaling |
| Reactome Nucleotide-binding domain leucine rich repeat containing receptor NLR signaling pathways | Genes involved in Nucleotide-binding domain. leucine rich repeat containing receptor (NLR) signaling pathways |
| Reactome Peptide ligand-binding receptors | Genes involved in Peptide ligand-binding receptors |
| Reactome regulation of IFNA signaling | Genes involved in Regulation of IFNA signaling |
| Reactome regulation of IFNG signaling | Genes involved in Regulation of IFNG signaling |
| Reactome RIG-I/MDA5 mediated induction of IFN-alpha/beta pathways | Genes involved in RIG-I/MDA5 mediated induction of IFN-alpha/beta pathways |
| Reactome signaling by interleukins | Genes involved in Signaling by Interleukins |
| Reactome the NLRP3 inflammasome | Genes involved in The NLRP3 inflammasome |
| Reactome TRAF3-dependent IRF activation pathway | Genes involved in TRAF3-dependent IRF activation pathway |
| Reactome TRAF6 mediated IRF7 activation | Genes involved in TRAF6 mediated IRF7 activation |
| Reactome Transcriptional regulation of white adipocyte differentiation | Genes involved in Transcriptional Regulation of White Adipocyte Differentiation |
| SA MMP cytokine connection | Cytokines can induce activation of matrix metalloproteinases. which degrade extracellular matrix. |
| ST IL-13 pathway | Interleukin 13 (IL-13) Pathway |
| ST Interferon gamma pathway | Interferon gamma pathway |
| ST Interleukin 4 pathway | Interleukin 4 (IL-4) Pathway |
| ST Tumor Necrosis Factor pathway | Tumor Necrosis Factor Pathway |
| ST Type I interferon pathway | Type I Interferon (alpha/beta IFN) Pathway |

**Supplementary Table 5: Results enrichment analyses of 162 inflammatory pathways per group of MDD patients as compared to controls**

|  | **All MDD** | | **MDD with hyperphagia** | | **MDD with hypophagia** | |
| --- | --- | --- | --- | --- | --- | --- |
| **Pathway** | *p* value | FDR | *p* value | FDR | *p* value | FDR |
| KEGG natural killer cell mediated cytotoxicity | 7.94E-6 | 1.29E-3 | 5.21E-4 | 0.017 | 0.014 | 0.186 |
| KEGG T cell receptor signaling pathway | 1.39E-4 | 7.52E-3 | 2.09E-3 | 0.034 | 0.041 | 0.222 |
| PID IL-8 CXCR2 pathway | 1.39E-4 | 7.52E-3 | 2.94E-3 | 0.041 | 5.79E-4 | 0.047 |
| Reactome adaptive immune system | 3.70E-4 | 0.015 | 4.25E-3 | 0.049 | 4.98E-4 | 0.047 |
| Reactome immune system | 5.60E-4 | 0.015 | 2.07E-3 | 0.034 | 3.98E-3 | 0.135 |
| PID IL-8 CXCR1 pathway | 5.71E-4 | 0.015 | 6.19E-3 | 0.049 | 2.32E-3 | 0.125 |
| Reactome signaling by interleukins | 6.93E-4 | 0.016 | 1.22E-3 | 0.033 | 0.034 | 0.221 |
| PID CXCR4 pathway | 1.41E-3 | 0.028 | 4.80E-3 | 0.049 | 7.66E-3 | 0.149 |
| Reactome IL-3, IL-5 and GM-CSF signaling | 2.80E-3 | 0.045 | 4.70E-4 | 0.017 | 8.10E-3 | 0.149 |
| Reactome apoptosis | 4.85E-3 | 0.071 | 0.015 | 0.094 | 0.010 | 0.149 |
| Reactome inflammasomes | 6.08E-3 | 0.082 | 1.71E-3 | 0.034 | 0.149 | 0.440 |
| BioCarta natural killer cells pathway | 2.62E-3 | 0.045 | 0.064 | 0.200 | 4.17E-3 | 0.135 |
| PID aMb2 neutrophils pathway | 7.69E-3 | 0.096 | 0.023 | 0.106 | 0.010 | 0.149 |
| PID CD8 TCR downstream pathway | 8.42E-3 | 0.097 | 0.028 | 0.112 | 0.038 | 0.221 |
| PID caspase pathway | 0.019 | 0.127 | 3.76E-4 | 0.017 | 0.056 | 0.274 |
| BioCarta GATA3 pathway | 0.028 | 0.159 | 4.10E-4 | 0.017 | 0.036 | 0.221 |
| PID NFAT transcription factor pathway | 0.030 | 0.160 | 5.10E-4 | 0.017 | 0.154 | 0.440 |
| BioCarta PAR1 pathway | 0.203 | 0.482 | 1.73E-3 | 0.034 | 0.127 | 0.398 |
| Reactome the NLRP3 inflammasome | 0.010 | 0.102 | 3.06E-3 | 0.041 | 0.024 | 0.221 |
| BioCarta IL22BP pathway | 0.075 | 0.296 | 4.11E-3 | 0.049 | 7.54E-3 | 0.149 |
| KEGG apoptosis | 0.045 | 0.209 | 5.10E-3 | 0.049 | 0.169 | 0.443 |
| PID IL-4 2pathway | 0.474 | 0.657 | 5.48E-3 | 0.049 | 0.170 | 0.443 |
| ST interleukin 4 pathway | 0.110 | 0.348 | 5.67E-3 | 0.049 | 0.010 | 0.149 |
| Reactome IL-6 signaling | 0.143 | 0.387 | 6.13E-3 | 0.049 | 0.107 | 0.367 |
| PID angiopoietin receptor pathway | 0.014 | 0.113 | 6.29E-3 | 0.049 | 0.028 | 0.221 |
| Reactome nucleotide-binding domain leucine-rich repeat containing receptor NLR signaling pathways | 0.052 | 0.225 | 7.73E-3 | 0.057 | 0.161 | 0.443 |
| Reactome cytokine signaling in immune system | 0.013 | 0.113 | 8.36E-3 | 0.059 | 0.128 | 0.398 |
| BioCarta IL2RB pathway | 0.123 | 0.353 | 0.013 | 0.084 | 0.208 | 0.487 |
| BioCarta mitochondria pathway | 0.303 | 0.572 | 0.016 | 0.094 | 0.224 | 0.505 |
| BioCarta IL-6 pathway | 0.497 | 0.657 | 0.016 | 0.094 | 0.182 | 0.452 |
| BioCarta TNFR1 pathway | 0.389 | 0.627 | 0.017 | 0.094 | 0.403 | 0.646 |
| KEGG JAK-STAT signaling pathway | 0.173 | 0.446 | 0.017 | 0.094 | 0.322 | 0.587 |
| Reactome extrinsic pathway for apoptosis | 0.015 | 0.114 | 0.017 | 0.094 | 0.308 | 0.584 |
| PID IL-2 STAT5 pathway | 0.137 | 0.377 | 0.018 | 0.095 | 0.180 | 0.452 |
| PID IL-12 2pathway | 0.021 | 0.133 | 0.019 | 0.096 | 0.037 | 0.221 |
| PID RXR/VDR pathway | 0.010 | 0.102 | 0.093 | 0.247 | 0.087 | 0.349 |
| BioCarta IL-7 pathway | 0.013 | 0.113 | 0.027 | 0.111 | 0.103 | 0.361 |
| KEGG leukocyte transendothelial migration | 0.014 | 0.113 | 0.122 | 0.286 | 0.017 | 0.188 |
| KEGG Fc epsilon RI signaling pathway | 0.017 | 0.126 | 0.166 | 0.332 | 0.031 | 0.221 |
| KEGG NOD-like receptor signaling pathway | 0.018 | 0.126 | 0.071 | 0.216 | 0.051 | 0.258 |
| PID CXCR3 pathway | 0.023 | 0.142 | 0.249 | 0.425 | 0.044 | 0.229 |
| SA MMP cytokine connection | 0.029 | 0.159 | 0.096 | 0.251 | 0.101 | 0.361 |
| Reactome chemokine receptors bind chemokines | 0.029 | 0.159 | 0.495 | 0.652 | 0.514 | 0.681 |
| BioCarta PML pathway | 0.031 | 0.160 | 0.245 | 0.425 | 0.020 | 0.203 |
| KEGG cytokine-cytokine receptor interaction | 0.036 | 0.181 | 0.212 | 0.396 | 0.723 | 0.819 |
| PID IL-12 STAT4 pathway | 0.037 | 0.181 | 0.172 | 0.335 | 0.427 | 0.646 |
| BioCarta FAS pathway | 0.045 | 0.209 | 0.028 | 0.112 | 0.039 | 0.221 |
| KEGG type I diabetes mellitus | 0.050 | 0.225 | 0.237 | 0.425 | 0.283 | 0.567 |
| KEGG chemokine signaling pathway | 0.053 | 0.225 | 0.124 | 0.286 | 0.093 | 0.359 |
| PID IL-1 pathway | 0.057 | 0.238 | 0.023 | 0.106 | 0.100 | 0.361 |
| Reactome IL-2 signaling | 0.060 | 0.241 | 0.046 | 0.157 | 0.034 | 0.221 |
| BioCarta granulocytes pathway | 0.084 | 0.315 | 0.574 | 0.693 | 0.187 | 0.452 |
| BioCarta Tob1 pathway | 0.084 | 0.315 | 0.284 | 0.446 | 0.258 | 0.544 |
| KEGG hematopoietic cell lineage | 0.087 | 0.317 | 0.570 | 0.693 | 0.566 | 0.717 |
| BioCarta longevity pathway | 0.088 | 0.317 | 0.122 | 0.286 | 0.187 | 0.452 |
| Reactome antiviral mechanism by IFN stimulated genes | 0.094 | 0.326 | 0.032 | 0.123 | 0.201 | 0.479 |
| BioCarta FCER1 pathway | 0.095 | 0.326 | 0.078 | 0.220 | 0.040 | 0.221 |
| PID IFNg pathway | 0.097 | 0.326 | 0.093 | 0.247 | 0.083 | 0.349 |
| BioCarta SODD pathway | 0.100 | 0.329 | 0.097 | 0.251 | 0.276 | 0.567 |
| KEGG type II diabetes mellitus | 0.105 | 0.339 | 0.516 | 0.653 | 0.250 | 0.539 |
| PID HDAC class I pathway | 0.113 | 0.352 | 0.298 | 0.459 | 0.076 | 0.343 |
| Reactome IL-1 signaling | 0.117 | 0.353 | 0.060 | 0.194 | 0.517 | 0.681 |
| Reactome IL receptor SHC signaling | 0.118 | 0.353 | 0.052 | 0.172 | 0.088 | 0.349 |
| BioCarta IL-4 pathway | 0.124 | 0.353 | 0.036 | 0.131 | 0.186 | 0.452 |
| Reactome hemostasis | 0.124 | 0.353 | 0.128 | 0.286 | 0.413 | 0.646 |
| BioCarta TALL-I pathway | 0.129 | 0.360 | 0.064 | 0.200 | 0.016 | 0.188 |
| BioCarta CXCR4 pathway | 0.166 | 0.434 | 0.035 | 0.131 | 0.371 | 0.644 |
| BioCarta IL-10 pathway | 0.166 | 0.434 | 0.040 | 0.143 | 0.408 | 0.646 |
| PID IL-27 pathway | 0.183 | 0.462 | 0.130 | 0.286 | 0.449 | 0.646 |
| BioCarta IL-17 pathway | 0.187 | 0.467 | 0.876 | 0.921 | 0.318 | 0.586 |
| BioCarta Thelper pathway | 0.203 | 0.482 | 0.824 | 0.884 | 0.228 | 0.506 |
| BioCarta fMLP pathway | 0.205 | 0.482 | 0.077 | 0.220 | 0.017 | 0.188 |
| KEGG proteasome | 0.207 | 0.482 | 0.381 | 0.551 | 0.025 | 0.221 |
| ST tumor necrosis factor pathway | 0.208 | 0.482 | 0.022 | 0.106 | 0.082 | 0.349 |
| BioCarta NTHI pathway | 0.217 | 0.493 | 0.323 | 0.489 | 0.073 | 0.343 |
| PID SHP2 pathway | 0.219 | 0.493 | 0.273 | 0.433 | 0.460 | 0.646 |
| BioCarta NKT pathway | 0.240 | 0.534 | 0.177 | 0.341 | 0.803 | 0.891 |
| KEGG antigen processing and presentation | 0.244 | 0.534 | 0.488 | 0.648 | 0.433 | 0.646 |
| Reactome IL-7 signaling | 0.249 | 0.538 | 0.158 | 0.327 | 0.481 | 0.661 |
| BioCarta IL1R pathway | 0.256 | 0.545 | 0.162 | 0.327 | 0.124 | 0.398 |
| BioCarta free pathway | 0.261 | 0.545 | 0.249 | 0.425 | 0.678 | 0.790 |
| BioCarta EPONFKB pathway | 0.263 | 0.545 | 0.273 | 0.433 | 0.146 | 0.437 |
| BioCarta Th1/Th2 pathway | 0.266 | 0.545 | 0.395 | 0.566 | 0.948 | 0.980 |
| BioCarta HSP27 pathway | 0.281 | 0.564 | 0.456 | 0.633 | 0.393 | 0.646 |
| BioCarta DC pathway | 0.283 | 0.564 | 0.247 | 0.425 | 0.556 | 0.710 |
| PID IL-2 1pathway | 0.286 | 0.564 | 0.048 | 0.163 | 0.033 | 0.221 |
| BioCarta Tid pathway | 0.300 | 0.572 | 0.467 | 0.636 | 0.096 | 0.361 |
| PID anthrax pathway | 0.300 | 0.572 | 0.819 | 0.884 | 0.293 | 0.578 |
| BioCarta NF-kB pathway | 0.301 | 0.572 | 0.147 | 0.309 | 0.282 | 0.567 |
| BioCarta p27 pathway | 0.316 | 0.579 | 0.168 | 0.332 | 0.457 | 0.646 |
| NABA matrisome associated | 0.320 | 0.579 | 0.909 | 0.931 | 0.941 | 0.980 |
| BioCarta IL-2 pathway | 0.321 | 0.579 | 0.041 | 0.143 | 0.140 | 0.429 |
| BioCarta CCR5 pathway | 0.322 | 0.579 | 0.102 | 0.258 | 0.155 | 0.440 |
| PID TCR calcium pathway | 0.328 | 0.584 | 0.130 | 0.286 | 0.525 | 0.685 |
| KEGG MAPK signaling pathway | 0.333 | 0.587 | 0.712 | 0.807 | 0.237 | 0.520 |
| BioCarta RNA pathway | 0.342 | 0.587 | 0.607 | 0.713 | 0.411 | 0.646 |
| Reactome interferon signaling | 0.343 | 0.587 | 0.123 | 0.286 | 0.314 | 0.584 |
| KEGG intestinal immune network for IGA production | 0.345 | 0.587 | 0.261 | 0.433 | 0.953 | 0.980 |
| BioCarta 41bb pathway | 0.348 | 0.587 | 0.121 | 0.286 | 0.165 | 0.443 |
| PID PDGFR-α pathway | 0.355 | 0.593 | 0.268 | 0.433 | 0.477 | 0.661 |
| BioCarta IL-12 pathway | 0.362 | 0.594 | 0.415 | 0.585 | 0.312 | 0.584 |
| PID telomerase pathway | 0.363 | 0.594 | 0.191 | 0.360 | 0.076 | 0.343 |
| PID ATF2 pathway | 0.391 | 0.627 | 0.295 | 0.459 | 0.216 | 0.493 |
| BioCarta Barrestin SRC pathway | 0.395 | 0.628 | 0.340 | 0.501 | 0.437 | 0.646 |
| NABA matrisome | 0.402 | 0.632 | 0.851 | 0.907 | 0.990 | 0.990 |
| BioCarta monocyte pathway | 0.420 | 0.634 | 0.512 | 0.653 | 0.435 | 0.646 |
| PID TNF pathway | 0.420 | 0.634 | 0.465 | 0.636 | 0.684 | 0.792 |
| BioCarta eryth pathway | 0.422 | 0.634 | 0.881 | 0.921 | 0.572 | 0.718 |
| Reactome G alpha I signaling events | 0.423 | 0.634 | 0.528 | 0.663 | 0.693 | 0.796 |
| BioCarta rela pathway | 0.426 | 0.634 | 0.141 | 0.301 | 0.455 | 0.646 |
| PID IL-5 pathway | 0.431 | 0.634 | 0.217 | 0.399 | 0.283 | 0.567 |
| KEGG TGF beta signaling pathway | 0.434 | 0.634 | 0.577 | 0.693 | 0.311 | 0.584 |
| KEGG toll like receptor signaling pathway | 0.437 | 0.634 | 0.088 | 0.244 | 0.883 | 0.947 |
| PID IL-6 7 pathway | 0.438 | 0.634 | 0.025 | 0.109 | 0.109 | 0.367 |
| PID IL-23 pathway | 0.443 | 0.636 | 0.075 | 0.220 | 0.447 | 0.646 |
| PID NF-kappaB canonical pathway | 0.460 | 0.654 | 0.092 | 0.247 | 0.501 | 0.676 |
| BioCarta GH pathway | 0.479 | 0.657 | 0.190 | 0.360 | 0.088 | 0.349 |
| Reactome TRAF3-dependent IRF activation pathway | 0.482 | 0.657 | 0.765 | 0.837 | 0.213 | 0.492 |
| BioCarta toll pathway | 0.488 | 0.657 | 0.220 | 0.400 | 0.432 | 0.646 |
| BioCarta IL-3 pathway | 0.494 | 0.657 | 0.122 | 0.286 | 0.039 | 0.221 |
| BioCarta NO2 IL-12 pathway | 0.494 | 0.657 | 0.504 | 0.653 | 0.628 | 0.770 |
| PID REG GR pathway | 0.502 | 0.657 | 0.027 | 0.111 | 0.557 | 0.710 |
| BioCarta lym pathway | 0.502 | 0.657 | 0.731 | 0.817 | 0.609 | 0.753 |
| NABA secreted factors | 0.503 | 0.657 | 0.957 | 0.963 | 0.986 | 0.990 |
| BioCarta IL-5 pathway | 0.515 | 0.667 | 0.400 | 0.569 | 0.440 | 0.646 |
| BioCarta asb cell pathway | 0.541 | 0.696 | 0.488 | 0.648 | 0.675 | 0.790 |
| Reactome regulation of IFNg signaling | 0.573 | 0.719 | 0.594 | 0.705 | 0.438 | 0.646 |
| PID SMAD2/3 nuclear pathway | 0.576 | 0.719 | 0.025 | 0.109 | 0.336 | 0.605 |
| BioCarta lair pathway | 0.576 | 0.719 | 0.695 | 0.798 | 0.595 | 0.741 |
| PID IL-3 pathway | 0.577 | 0.719 | 0.162 | 0.327 | 0.120 | 0.397 |
| ST interferon gamma pathway | 0.587 | 0.726 | 0.338 | 0.501 | 0.463 | 0.646 |
| PID IL-2 pi3k pathway | 0.617 | 0.758 | 0.268 | 0.433 | 0.821 | 0.899 |
| Reactome interferon gamma signaling | 0.644 | 0.782 | 0.500 | 0.653 | 0.358 | 0.630 |
| BioCarta cytokine pathway | 0.647 | 0.782 | 0.572 | 0.693 | 0.678 | 0.790 |
| Reactome transcriptional regulation of white adipocyte differentiation | 0.675 | 0.810 | 0.750 | 0.833 | 0.549 | 0.710 |
| ST IL-13 pathway | 0.687 | 0.819 | 0.073 | 0.220 | 0.432 | 0.646 |
| BioCarta stress pathway | 0.699 | 0.825 | 0.510 | 0.653 | 0.379 | 0.644 |
| PID AP1 pathway | 0.703 | 0.825 | 0.130 | 0.286 | 0.311 | 0.584 |
| Reactome innate immune system | 0.712 | 0.830 | 0.333 | 0.500 | 0.654 | 0.785 |
| Reactome RIG-I/MDA5 mediated induction of IFN-alpha/beta pathways | 0.719 | 0.832 | 0.322 | 0.489 | 0.382 | 0.644 |
| PID ceramide pathway | 0.729 | 0.838 | 0.252 | 0.426 | 0.654 | 0.785 |
| BioCarta PPARA pathway | 0.744 | 0.841 | 0.480 | 0.648 | 0.258 | 0.544 |
| BioCarta stem pathway | 0.746 | 0.841 | 0.725 | 0.816 | 0.513 | 0.681 |
| BioCarta p38MAPK pathway | 0.747 | 0.841 | 0.893 | 0.922 | 0.159 | 0.443 |
| KEGG adipocytokine signaling pathway | 0.773 | 0.864 | 0.562 | 0.693 | 0.718 | 0.819 |
| BioCarta MAPK pathway | 0.783 | 0.868 | 0.971 | 0.971 | 0.356 | 0.630 |
| BioCarta CD40 pathway | 0.804 | 0.879 | 0.643 | 0.749 | 0.632 | 0.770 |
| BioCarta CDMAC pathway | 0.806 | 0.879 | 0.263 | 0.433 | 0.433 | 0.646 |
| Reactome peptide ligand binding receptors | 0.808 | 0.879 | 0.914 | 0.931 | 0.897 | 0.950 |
| ST Type I interferon pathway | 0.833 | 0.895 | 0.135 | 0.291 | 0.800 | 0.891 |
| BioCarta GCR pathway | 0.834 | 0.895 | 0.457 | 0.633 | 0.379 | 0.644 |
| BioCarta inflam pathway | 0.845 | 0.901 | 0.658 | 0.761 | 0.780 | 0.878 |
| KEGG rig I like receptor signaling pathway | 0.851 | 0.901 | 0.377 | 0.550 | 0.492 | 0.669 |
| KEGG complement and coagulation cascades | 0.890 | 0.935 | 0.880 | 0.921 | 0.817 | 0.899 |
| BioCarta CCR3 pathway | 0.897 | 0.935 | 0.240 | 0.425 | 0.862 | 0.931 |
| KEGG cytosolic DNA sensing pathway | 0.900 | 0.935 | 0.596 | 0.705 | 0.861 | 0.931 |
| BioCarta classic pathway | 0.929 | 0.958 | 0.950 | 0.962 | 0.988 | 0.990 |
| Reactome TRAF6-mediated IRF7 activation | 0.938 | 0.962 | 0.712 | 0.807 | 0.665 | 0.790 |
| Reactome Interferon alpha/beta signaling | 0.957 | 0.975 | 0.756 | 0.833 | 0.956 | 0.980 |
| Reactome regulation of IFNa signaling | 0.965 | 0.977 | 0.572 | 0.693 | 0.948 | 0.980 |
| Reactome factors involved in megakaryocyte development and platelet production | 0.984 | 0.990 | 0.892 | 0.922 | 0.978 | 0.990 |
| KEGG regulation of autophagy | 0.992 | 0.992 | 0.776 | 0.844 | 0.892 | 0.950 |

The upper 35 pathways are enriched at an FDR<0.1 in at least one group. The lower 127 pathways are not significantly enriched in any group.

**Supplementary Table 6: Percentage of the 25% percentile genes with a positive effect size in the differential gene expression analysis, for each of the 35 enriched (FDR<0.1) pathway separately**

|  | **N genes in pathway** | **Percentage of genes with positive beta compared to controls** | | |
| --- | --- | --- | --- | --- |
| **Pathway** |  | All MDD | MDD with hyperphagia | MDD with hypophagia |
| KEGG natural killer cell mediated cytotoxicity | 122 | 35.5 | 35.5 | 38.7 |
| KEGG T cell receptor signaling | 108 | 37 | 25.9 | 37 |
| PID IL-8 CXCR2 | 34 | 100 | 77.8 | 100 |
| Reactome adaptive immune system | 483 | 32.2 | 42.1 | 29.8 |
| Reactome immune system | 840 | 45.2 | 51.9 | 43.3 |
| PID IL-8 CXCR1 | 28 | 100 | 71.4 | 100 |
| Reactome signaling by interleukins | 102 | 57.7 | 50 | 65.4 |
| PID CXCR4 pathway | 101 | 60 | 60 | 72 |
| Reactome IL-3, IL-5 and GM-CSF signaling | 40 | 50 | 40 | 60 |
| Reactome apoptosis | 136 | 35.3 | 47.1 | 26.5 |
| Reactome inflammasomes | 16 | 75 | 75 | 100 |
| BioCarta natural killer cells pathway | 19 | 20 | 20 | 20 |
| PID aMb2 neutrophils pathway | 40 | 80 | 80 | 90 |
| PID CD8 TCR downstream pathway | 63 | 18.8 | 31.3 | 25 |
| PID caspase pathway | 50 | 53.8 | 46.2 | 30.8 |
| BioCarta GATA3 pathway | 16 | 25 | 50 | 25 |
| PID NFAT transcription factor pathway | 47 | 16.7 | 25 | 41.7 |
| BioCarta PAR1 pathway | 37 | 66.7 | 55.6 | 33.3 |
| Reactome the NLRP3 inflammasome | 11 | 100 | 66.7 | 100 |
| BioCarta IL22BP pathway | 16 | 50 | 75 | 75 |
| KEGG apoptosis | 85 | 52.4 | 47.6 | 38.1 |
| PID IL-4 2pathway | 61 | 66.7 | 66.7 | 80 |
| ST interleukin 4 pathway | 26 | 57.1 | 71.4 | 71.4 |
| Reactome IL-6 signaling | 10 | 100 | 100 | 100 |
| PID angiopoietin receptor pathway | 50 | 53.8 | 69.2 | 61.5 |
| Reactome nucleotide-binding domain leucine-rich repeat containing receptor NLR signaling pathways | 44 | 54.5 | 54.5 | 54.5 |
| Reactome cytokine signaling in immune system | 247 | 41.9 | 45.2 | 53.2 |
| BioCarta IL2RB pathway | 38 | 40 | 50 | 60 |
| BioCarta mitochondria pathway | 19 | 20 | 60 | 0 |
| BioCarta IL-6 pathway | 22 | 66.7 | 66.7 | 66.7 |
| BioCarta TNFR1 pathway | 29 | 71.4 | 85.7 | 57.1 |
| KEGG JAK-STAT signaling pathway | 145 | 58.3 | 63.9 | 63.9 |
| Reactome extrinsic pathway for apoptosis | 13 | 33.3 | 66.7 | 66.7 |
| PID IL-2 STAT5 pathway | 30 | 25 | 25 | 37.5 |
| PID IL-12 2pathway | 62 | 31.3 | 43.8 | 31.3 |

This table presents, for each enriched pathway, the percentage of top 25% genes with a positive effect size in the differential gene expression. The column with number of genes present in pathway represents the number of genes in that pathway measured by the microarrays used in the NESDA dataset.

| **Supplementary Table 7: 32 canonical pathways enriched at FDR<0.1 in at least one group of MDD patients as compared to controls in overall enrichment analyses including all 1 329 canonical pathways** | | | | |
| --- | --- | --- | --- | --- |
|  | ***P* value** | | | |
| **Pathway** | All MDD | MDD with hyperphagia | MDD with hypophagia |  |
| KEGG natural killer cell mediated cytotoxicity | **7.94E-06*** | **5.21E-04*** | 0.014 |  |
| ST T cell signal transduction | **2.31E-05** | 0.011 | 0.017 |  |
| PID retinoic acid pathway | **3.83E-05** | 4.20E-03 | 5.00E-04 |  |
| PID IL-8 CXCR2 pathway | **1.39E-04*** | 2.94E-03 | 5.79E-04 |  |
| KEGG T cell receptor signaling pathway | **1.39E-04*** | 2.09E-03 | 0.041 |  |
| KEGG Parkinson’s disease | **2.32E-04** | 0.032 | 0.029 |  |
| Reactome immunoregulatory interactions between a lymphoid and a non-lymphoid cell | **2.57E-04** | 0.218 | 9.91E-03 |  |
| KEGG viral myocarditis | **3.02E-04** | 0.053 | 0.025 |  |
| Reactome adaptive immune system | **3.70E-04*** | 4.25E-03 | 4.98E-04 |  |
| Reactome regulation of signaling by Cbl | **4.97E-04** | **3.18E-04** | 0.030 |  |
| Reactome signaling by SCF-KIT | **5.35E-04** | 0.017 | 2.65E-03 |  |
| Reactome immune system | **5.60E-04*** | 2.07E-03 | 3.98E-03 |  |
| PID IL-8 CXCR1 pathway | **5.71E-04*** | 6.19E-03 | 2.32E-03 |  |
| Reactome signaling by interleukins | **6.93E-04*** | 1.22E-03 | 0.034 |  |
| Reactome respiratory electron transport | **6.96E-04** | 0.429 | 0.017 |  |
| PID FoxO pathway | **7.18E-04** | 5.28E-03 | 2.09E-04 |  |
| SIG BCR signaling pathway | **7.44E-04** | 0.031 | 1.85E-03 |  |
| Reactome TCR signaling | **7.66E-04** | 0.080 | 0.207 |  |
| PID CD8 TCR downstream pathway | **8.33E-04*** | 0.119 | 0.096 |  |
| Reactome glycerophospholipid biosynthesis | **8.71E-04** | 0.031 | 0.011 |  |
| BioCarta D4-GDI pathway | **8.95E-04** | **5.52E-04** | 0.010 |  |
| Reactome processing of capped intron containing pre-mRNA | **9.73E-04** | **2.88E-04** | 6.83E-04 |  |
| PID NFAT 3pathway | **1.07E-03** | 0.017 | 0.024 |  |
| PID CXCR4 pathway | **1.41E-03*** | 4.80E-03 | 7.66E-03 |  |
| KEGG pentose phosphate pathway | **1.46E-03** | 3.09E-03 | 4.03E-03 |  |
| Reactome generation of second messenger molecules | **1.50E-03** | 0.173 | 0.195 |  |
| Reactome mRNA splicing minor pathway | **2.01E-03** | 7.83E-03 | 4.47E-03 |  |
| BioCarta caspase pathway | 3.19E-03 | **7.62E-05** | 5.15E-03 |  |
| PID caspase pathway | 0.019 | **3.76E-04*** | 0.056 |  |
| BioCarta GATA3 pathway | 0.028 | **4.10E-04*** | 0.036 |  |
| Reactome IL-3, IL-5 and GM-CSF signaling | 2.80E-03 | **4.70E-04*** | 8.10E-03 |  |
| PID NFAT transcription factor pathway | 0.030 | **5.10E-04*** | 0.154 |  |

The values represent the raw *p* values obtained from the enrichment analyses. Bold values correspond to an FDR<0.1. * Indicates that the pathways are part of the 162 inflammatory pathways we selected.

**Supplementary Table 8: Results of enrichment analysis of 162 inflammatory pathways after adjustment for BMI in current MDD patients with hyperphagia as compared to controls**

|  | **MDD with hyperphagia** | |
| --- | --- | --- |
| **Pathway** | *p* value | FDR |
| PID NFAT transcription factor pathway | 1.81E-03* | 0.099 |
| PID IL-8 CXCR2 pathway | 2.09E-03* | 0.099 |
| PID caspase pathway | 2.23E-03* | 0.099 |
| PID CXCR4 pathway | 2.97E-03* | 0.099 |
| BioCarta GATA3 pathway | 3.07E-03* | 0.099 |
| KEGG Natural killer cell mediated cytotoxicity | 3.85E-03* | 0.104 |
| PID IL-8 CXCR1 pathway | 5.71E-03* | 0.116 |
| KEGG T cell receptor signaling pathway | 5.75E-03* | 0.116 |
| Reactome The NLRP3 inflammasome | 6.67E-03* | 0.119 |
| Reactome IL-3, IL-5 and GM-CSF signaling | 7.33E-03* | 0.119 |
| Reactome Inflammasomes | 0.012* | 0.178 |
| KEGG apoptosis | 0.015* | 0.203 |
| BioCarta PAR1 pathway | 0.025* | 0.258 |
| KEGG chemokine signaling pathway | 0.025* | 0.258 |
| Reactome signaling by interleukins | 0.028* | 0.258 |
| PID angiopoietin receptor pathway | 0.028* | 0.258 |
| PID IL-1 pathway | 0.031 | 0.258 |
| BioCarta mitochondria pathway | 0.032* | 0.258 |
| PID RXR/VDR pathway | 0.032 | 0.258 |
| ST Interleukin 4 pathway | 0.032* | 0.258 |
| Reactome Adaptive immune system | 0.038* | 0.291 |
| BioCarta Natural killer cells pathway | 0.040* | 0.297 |
| Reactome Nucleotide-binding domain leucine rich repeat containing receptor NLR signaling pathways | 0.043* | 0.305 |
| PID aMb2 neutrophils pathway | 0.046* | 0.308 |
| PID CXCR3 pathway | 0.063 | 0.398 |
| BioCarta IL2RB pathway | 0.066 | 0.398 |
| PID CD8 TCR downstream pathway | 0.068 | 0.398 |
| PID IL-12 2pathway | 0.069 | 0.398 |
| KEGG Leukocyte transendothelial migration | 0.075 | 0.417 |
| BioCarta FAS pathway | 0.078 | 0.417 |
| BioCarta TALL1 pathway | 0.080 | 0.417 |
| BioCarta IL22BP pathway | 0.086 | 0.426 |
| Reactome Extrinsic pathway for apoptosis | 0.094 | 0.426 |
| Reactome IL-6 signaling | 0.094 | 0.426 |
| BioCarta 41BB pathway | 0.096 | 0.426 |
| BioCarta TNFR1 pathway | 0.099 | 0.426 |
| BioCarta CXCR4 pathway | 0.104 | 0.426 |
| ST Tumor Necrosis Factor pathway | 0.107 | 0.426 |
| KEGG NOD-like receptor signaling pathway | 0.107 | 0.426 |
| KEGG JAK-STAT signaling pathway | 0.109 | 0.426 |
| PID REG GR pathway | 0.117 | 0.426 |
| Reactome Hemostasis | 0.117 | 0.426 |
| PID IL-4 2pathway | 0.118 | 0.426 |
| Reactome Apoptosis | 0.119 | 0.426 |
| PID IL-6 7 pathway | 0.120 | 0.426 |
| SA MMP Cytokine connection | 0.121 | 0.426 |
| BioCarta IL-6 pathway | 0.128 | 0.441 |
| Reactome Immune system | 0.139 | 0.453 |
| BioCarta RELA pathway | 0.140 | 0.453 |
| BioCarta IL-10 pathway | 0.142 | 0.453 |
| PID IL-2 1pathway | 0.142 | 0.453 |
| Reactome IL-2 signaling | 0.151 | 0.466 |
| BioCarta fMLP pathway | 0.156 | 0.466 |
| PID IL-2 STAT5 pathway | 0.156 | 0.466 |
| BioCarta IL-2 pathway | 0.161 | 0.466 |
| BioCarta FCER1 pathway | 0.162 | 0.466 |
| Reactome cytokine signaling in immune system | 0.164 | 0.466 |
| BioCarta SODD pathway | 0.170 | 0.474 |
| Reactome IL-1 signaling | 0.176 | 0.483 |
| BioCarta CCR5 pathway | 0.186 | 0.503 |
| Reactome antiviral mechanism by IFN stimulated genes | 0.195 | 0.514 |
| Reactome IL receptor SHC signaling | 0.197 | 0.514 |
| BioCarta GCR pathway | 0.202 | 0.519 |
| BioCarta IL-4 pathway | 0.206 | 0.521 |
| BioCarta NF-kB pathway | 0.209 | 0.521 |
| BioCarta HSP27 pathway | 0.213 | 0.524 |
| PID IL-23 pathway | 0.219 | 0.531 |
| PID AP1 pathway | 0.248 | 0.575 |
| KEGG Fc epsilon RI signaling pathway | 0.254 | 0.575 |
| BioCarta IL-3 pathway | 0.256 | 0.575 |
| PID HDAC class I pathway | 0.259 | 0.575 |
| Reactome G alpha I signaling events | 0.262 | 0.575 |
| BioCarta PML pathway | 0.262 | 0.575 |
| BioCarta FREE pathway | 0.263 | 0.575 |
| BioCarta P27 pathway | 0.274 | 0.591 |
| BioCarta Longevity pathway | 0.282 | 0.602 |
| PID TCR Calcium pathway | 0.317 | 0.661 |
| PID NF-kappaB canonical pathway | 0.322 | 0.661 |
| BioCarta IL-7 pathway | 0.323 | 0.661 |
| PID Telomerase pathway | 0.333 | 0.661 |
| BioCarta GH pathway | 0.334 | 0.661 |
| BioCarta Monocyte pathway | 0.338 | 0.661 |
| PID IFNg pathway | 0.339 | 0.661 |
| PID IL-12 STAT4 pathway | 0.348 | 0.671 |
| KEGG Toll-like receptor signaling pathway | 0.357 | 0.671 |
| BioCarta IL1R pathway | 0.363 | 0.671 |
| PID PDGFRA pathway | 0.363 | 0.671 |
| PID IL-3 pathway | 0.372 | 0.671 |
| PID SHP2 pathway | 0.377 | 0.671 |
| BioCarta EPONFKB pathway | 0.377 | 0.671 |
| KEGG Cytokine-cytokine receptor interaction | 0.380 | 0.671 |
| BioCarta IL-5 pathway | 0.381 | 0.671 |
| BioCarta LAIR pathway | 0.397 | 0.686 |
| BioCarta PPARA pathway | 0.399 | 0.686 |
| BioCarta DC pathway | 0.402 | 0.686 |
| Reactome Chemokine receptors bind chemokines | 0.410 | 0.686 |
| BioCarta CDMAC pathway | 0.411 | 0.686 |
| BioCarta CCR3 pathway | 0.420 | 0.690 |
| PID IL-5 pathway | 0.421 | 0.690 |
| BioCarta Barrestin SRC pathway | 0.437 | 0.691 |
| KEGG Type I diabetes mellitus | 0.439 | 0.691 |
| Reactome RIG-I/MDA5 mediated induction of IFN-alpha/beta pathways | 0.440 | 0.691 |
| KEGG Proteasome | 0.440 | 0.691 |
| ST Type I interferon pathway | 0.444 | 0.691 |
| BioCarta asb cell pathway | 0.453 | 0.699 |
| KEGG Cytosolic DNA-sensing pathway | 0.460 | 0.702 |
| KEGG RIG-I-like receptor signaling pathway | 0.476 | 0.721 |
| PID SMAD2/3 nuclear pathway | 0.494 | 0.741 |
| ST IL-13 pathway | 0.506 | 0.750 |
| Reactome Interferon signaling | 0.511 | 0.750 |
| BioCarta TOLL pathway | 0.514 | 0.750 |
| PID Ceramide pathway | 0.523 | 0.756 |
| BioCarta Cytokine pathway | 0.541 | 0.776 |
| PID IL-2 PI3K pathway | 0.565 | 0.803 |
| KEGG Type II diabetes mellitus | 0.571 | 0.804 |
| KEGG Intestinal immune network for IgA production | 0.613 | 0.847 |
| BioCarta NKT pathway | 0.614 | 0.847 |
| BioCarta NTHI pathway | 0.617 | 0.847 |
| PID IL-27 pathway | 0.622 | 0.847 |
| KEGG Antigen processing and presentation | 0.637 | 0.860 |
| PID TNF pathway | 0.645 | 0.863 |
| BioCarta Granulocytes pathway | 0.655 | 0.863 |
| BioCarta CD40 pathway | 0.656 | 0.863 |
| PID ATF2 pathway | 0.664 | 0.864 |
| BioCarta Lym pathway | 0.667 | 0.864 |
| BioCarta Tid pathway | 0.675 | 0.867 |
| BioCarta IL-12 pathway | 0.680 | 0.867 |
| ST Interferon gamma pathway | 0.685 | 0.868 |
| KEGG Complement and coagulation cascades | 0.703 | 0.871 |
| KEGG MAPK Signaling pathway | 0.719 | 0.871 |
| Reactome Innate immune system | 0.720 | 0.871 |
| BioCarta Th1/Th2 pathway | 0.720 | 0.871 |
| Reactome TRAF3-dependent IRF activation pathway | 0.722 | 0.871 |
| KEGG Hematopoietic cell lineage | 0.724 | 0.871 |
| Reactome Transcriptional regulation of white adipocyte differentiation | 0.726 | 0.871 |
| Reactome IL-7 signaling | 0.735 | 0.876 |
| BioCarta Stem pathway | 0.751 | 0.883 |
| Reactome TRAF6 mediated IRF7 activation | 0.754 | 0.883 |
| BioCarta T-helper pathway | 0.758 | 0.883 |
| BioCarta RNA pathway | 0.777 | 0.893 |
| KEGG TGF-beta signaling pathway | 0.778 | 0.893 |
| BioCarta Stress pathway | 0.790 | 0.896 |
| BioCarta NO2 IL-12 pathway | 0.791 | 0.896 |
| KEGG Adipocytokine signaling pathway | 0.797 | 0.897 |
| BioCarta TOB1 pathway | 0.803 | 0.897 |
| Reactome Regulation of IFNA signaling | 0.809 | 0.898 |
| BioCarta Inflam pathway | 0.821 | 0.905 |
| KEGG Regulation of Autophagy | 0.844 | 0.924 |
| Reactome Interferon gamma signaling | 0.860 | 0.935 |
| Reactome Interferon alpha/beta signaling | 0.909 | 0.972 |
| NABA Matrisome | 0.910 | 0.972 |
| Reactome Peptide ligand-binding receptors | 0.912 | 0.972 |
| BioCarta Classic pathway | 0.920 | 0.974 |
| NABA Matrisome associated | 0.929 | 0.977 |
| Reactome Regulation of IFNG signaling | 0.950 | 0.982 |
| Reactome Factors involved in megakaryocyte development and platelet production | 0.964 | 0.982 |
| PID Anthrax pathway | 0.964 | 0.982 |
| NABA Secreted factors | 0.965 | 0.982 |
| BioCarta Eryth pathway | 0.969 | 0.982 |
| BioCarta p38 MAPK pathway | 0.969 | 0.982 |
| BioCarta IL-17 pathway | 0.976 | 0.983 |
| BioCarta MAPK pathway | 0.986 | 0.986 |

* indicates that these nominally significant *p* values were enriched in the initial enrichment analysis, without adjustment for BMI in the differential gene expression analysis.
