# Supplementary figures and images for: Involvement of inflammatory gene expression pathways in depressed patients with hyperphagia
Source: Transl Psychiatry. 2019 Aug 20;9:193. doi: 10.1038/s41398-019-0528-0 (PMC6702221; doi:10.1038/s41398-019-0528-0)

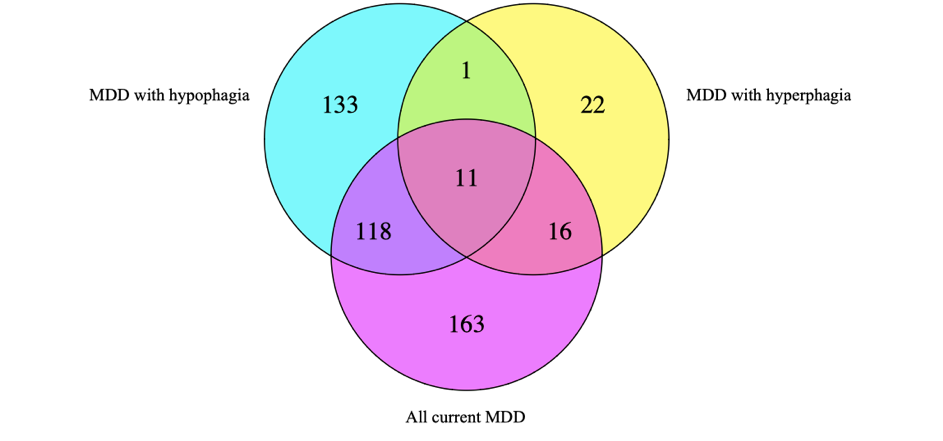

Supplement: Supplementary file 3 — Supplementary Figure 1: Number of differentially expressed genes per (sub)group of MDD cases at FDR<0.1 [file 41398_2019_528_MOESM3_ESM.tif]
